# Real-World Renal and Metabolic Outcomes of SGLT-2 Inhibitor Therapy in Patients with Type 2 Diabetes Mellitus

**DOI:** 10.3390/jcm15208076

**Published:** 2026-10-17

**Authors:** Öykü Ünsal, Hüseyin Döngelli, Mehmet Çağrı Ünal, Gökçen Güngör Semiz, Aysu Okumuş Güçlü, Tevfik Demir

**Affiliations:** 1 Department of Internal Medicine, Dokuz Eylul University Research and Training Hospital, 35330 Izmir, Turkey; huseyin.dongelli@deu.edu.tr (H.D.); aysuokumus@gmail.com (A.O.G.); 2 Division of Endocrinology and Metabolism, Department of Internal Medicine, Dokuz Eylul University Research and Training Hospital, 35330 Izmir, Turkey; munalcagri@gmail.com (M.Ç.Ü.);

**Keywords:** sodium–glucose co-transporter 2 inhibitors, type 2 diabetes mellitus, albuminuria, estimated glomerular filtration rate, real-world evidence, diabetic kidney disease

## Abstract

**Background/Objectives:** Real-world data are limited for short-term renal and metabolic changes after the initiation of sodium–glucose co-transporter 2 (SGLT2) inhibitors in Turkish clinical practice. We described 1-year changes in these parameters in adults with type 2 diabetes who started treatment with empagliflozin or dapagliflozin. **Methods:** In this single-centre retrospective cohort (2016–2021; *n* = 780), paired baseline and 12-month values were compared in patients with available measurements. The urinary albumin-to-creatinine ratio (UACR) was compared with the Wilcoxon signed-rank test. Factors associated with albuminuria response (≥30% UACR reduction) were examined using multivariable logistic regression. **Results:** HbA1c decreased by 1.0% (95% CI 0.8–1.1; *n* = 398), haematocrit increased by 1.3 percentage points (*n* = 418), uric acid decreased by 0.5 mg/dL (*n* = 221) and eGFR declined by 3.2 mL/min/1.73 m^2^ (95% CI 2.1–4.2; *n* = 426). In 109 patients with paired UACR, the median UACR fell from 20.9 [9.0–49.0] to 11.0 [6.0–30.0] mg/g (*p* < 0.001). A ≥30% reduction occurred in 81.1% of patients with a baseline UACR ≥30 mg/g and 41.7% of patients with a baseline UACR <30 mg/g (adjusted odds ratio: 6.10; 95% CI: 2.24–16.57). Genitourinary infections were more frequent in women as the most common documented reason for discontinuation. **Conclusions:** In routine care, SGLT2 inhibitor initiation was followed by changes in glycaemic, haematological, uric acid and albuminuria parameters that were similar in direction to those reported in randomised trials; the uncontrolled design and incomplete UACR data preclude causal inference.

## 1. Introduction

Diabetes mellitus affects an estimated 589 million adults aged 20–79 years worldwide, a number projected to reach 853 million by 2050; in Türkiye, the estimated age-standardised prevalence among adults is 16.5% [1]. Diabetic kidney disease (DKD) develops in approximately 40% of people with diabetes and is a leading cause of kidney failure and an important contributor to cardiovascular morbidity and mortality [2]. Albuminuria and reduced estimated glomerular filtration rate (eGFR) are independently associated with cardiovascular and kidney outcomes; the risk gradient associated with albuminuria is continuous, extending below the conventional 30 mg/g threshold [3]. Therefore, current guidelines recommend at least an annual measurement of the urinary albumin-to-creatinine ratio (UACR) and eGFR in people with type 2 diabetes mellitus (T2DM), together with kidney-protective therapy [4,5,6].

Sodium–glucose co-transporter 2 (SGLT2) inhibitors were developed as glucose-lowering agents but have since shown kidney- and cardiovascular-related benefits in large, randomised trials. In EMPA-REG OUTCOME, CANVAS, DECLARE-TIMI 58 and CREDENCE, which enrolled patients with T2DM, SGLT2 inhibition reduced cardiovascular events and slowed kidney disease progression [7,8,9,10,11]. DAPA-CKD and EMPA-KIDNEY extended kidney-related benefit to patients with chronic kidney disease (CKD) with and without diabetes [12,13]. Pre-specified and post hoc analyses of these trials showed that SGLT2 inhibitors reduce albuminuria relative to a placebo, with larger reductions in patients with higher baseline albuminuria [14,15,16,17]. A meta-analysis of randomised trials also confirmed a consistent albuminuria-lowering effect [18].

The proposed mechanisms include restoration of tubuloglomerular feedback through increased distal sodium delivery to the macula densa, leading to afferent arteriolar vasoconstriction and a reduction in intraglomerular pressure [19]. This haemodynamic effect is thought to explain the small, reversible decrease in eGFR typically observed within the first weeks of treatment (the initial eGFR “dip”); in trials, this is followed by a slower long-term decline than a placebo [20]. SGLT2 inhibitors also cause a sustained rise in haematocrit and a reduction in serum uric acid [21,22,23].

Randomised trials enrol selected populations; observational studies are therefore needed to describe changes in patient laboratory parameters in routine practice. Large international real-world studies such as CVD-REAL and EMPRISE have reported cardiovascular and heart failure outcomes associated with SGLT2 inhibitor use [24,25]; however, data remain limited for short-term changes in kidney and metabolic laboratory parameters in Turkish clinical practice. We therefore described changes in glycaemic, renal, haematological and metabolic parameters during the first year after empagliflozin or dapagliflozin treatment initiation in adults with T2DM followed at a university hospital in Türkiye and explored baseline factors associated with albuminuria response.

## 2. Materials and Methods

### 2.1. Study Design, Setting and Participants

**Figure 1:**
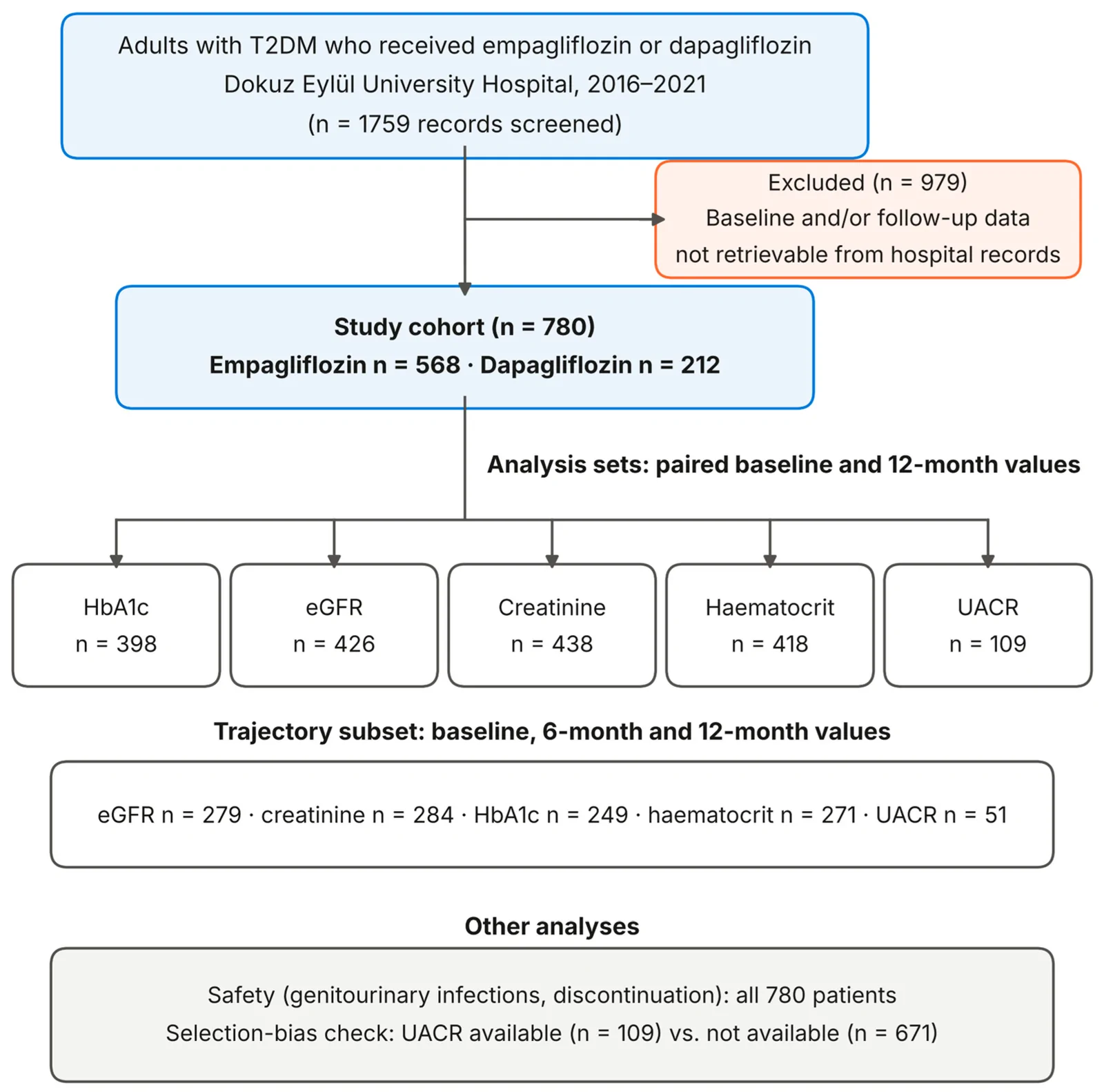
Flow of patients throughout the study and analysis sets. Overlap of analysis sets; each includes all patients with indicated paired measurements. Abbreviations: eGFR, estimated glomerular filtration rate; T2DM, type 2 diabetes mellitus; UACR, urinary albumin-to-creatinine ratio.

This retrospective cohort study was conducted at Dokuz Eylül University Hospital, a tertiary university hospital in İzmir, Türkiye, and is reported in accordance with the Strengthening the Reporting of Observational Studies in Epidemiology (STROBE) statement [26]. The hospital information system was searched for adults (≥18 years) with T2DM who received empagliflozin or dapagliflozin between January 2016 and December 2021; 1759 patients were identified. Patients whose demographic, clinical and laboratory data could be retrieved from the electronic records for the baseline visit and at least one follow-up visit were included (*n* = 780). The remaining 979 patients were excluded because these data could not be retrieved; for the same reason, their characteristics could not be compared with those of the included patients (Figure 1). No additional exclusion criteria based on comorbidity or kidney function were applied.

As a retrospective study of routine care, not all laboratory parameters were measured at every visit. Therefore, each analysis included all patients with the relevant paired measurements (available-case analysis), and the number of contributing patients is reported throughout (Figure 1). All patients included in the paired 12-month analyses had a documented SGLT2 inhibitor treatment duration of at least 12 months.

### 2.2. Data Collection and Definitions

Age, sex, diabetes duration, comorbidities (hypertension, hyperlipidaemia, coronary artery disease, heart failure, cerebrovascular disease, peripheral artery disease, CKD, malignancy and diabetic foot), glucose-lowering therapy at SGLT2 inhibitor initiation (metformin, sulfonylurea, pioglitazone, dipeptidyl peptidase-4 inhibitor, glucagon-like peptide-1 receptor agonist and insulin), the SGLT2 inhibitor agent, treatment discontinuation and the reason behind it, and genitourinary infections were extracted from outpatient notes and prescription records. Comorbidities, including CKD, were recorded as documented diagnoses in the medical history.

The following laboratory values recorded at baseline (SGLT2 inhibitor initiation), 6 months and 12 months were extracted: HbA1c, fasting plasma glucose, serum creatinine, eGFR, UACR, haematocrit, low-density lipoprotein cholesterol (LDL-C), triglycerides, uric acid, sodium and potassium. Body mass index (BMI) values were extracted when recorded. One value per time point was used. eGFR values were reported by the hospital laboratory; agreement between the reported values and those recalculated from serum creatinine, age and sex confirmed that the laboratory used the 2009 Chronic Kidney Disease Epidemiology Collaboration (CKD-EPI) creatinine equation [27]. Physiologically implausible values inconsistent with other measurements from the same patient and attributable to data-entry errors (26 values in 13 patients; for example, eGFR values equal to the concurrent serum creatinine value) were set to missing before analysis; no values were imputed.

Albuminuria was categorised according to KDIGO as A1 (<30 mg/g), A2 (30–300 mg/g) or A3 (>300 mg/g) [6]. The primary albuminuria outcome, albuminuria response, was defined as a reduction in UACR of at least 30% from baseline to 12 months, a threshold commonly used in trials and associated with subsequent kidney outcomes [28]. Regression to a lower KDIGO category was reported as a secondary descriptive outcome. Genitourinary infections were defined as clinically diagnosed and treated urinary tract or genital mycotic infections, as documented in outpatient notes and prescription records during SGLT2 inhibitor treatment; culture confirmation was not systematically available.

### 2.3. Statistical Analysis

The original descriptive analyses were performed using IBM SPSS Statistics version 22.0 (IBM Corp., Armonk, NY, USA); all analyses reported here were performed on the same dataset with Python version 3.13 (SciPy 1.18 and scikit-learn 1.9). Continuous variables are presented as mean ± standard deviation (SD), and variables with skewed distributions (UACR, serum creatinine, and triglycerides at baseline) as the median [interquartile range, IQR]; categorical variables are presented as *n* (%). Changes from baseline to 12 months are reported as mean differences with 95% confidence intervals (CIs) and compared with the paired *t*-test; the Wilcoxon signed-rank test was used as a sensitivity analysis for all parameters and as the primary test for UACR. For UACR, the geometric mean ratio (12 months/baseline) was also estimated from log-transformed values. Changes across baseline, 6 and 12 months were assessed in patients with all three measurements using the Friedman test, with paired *t*-tests for pairwise comparisons.

Patients with and without paired UACR data, and those treated with empagliflozin and dapagliflozin, were compared using Welch’s *t*-test or the Mann–Whitney U test for continuous variables and the chi-squared or Fisher’s exact test for categorical variables. The association between changes in UACR and eGFR was assessed with Spearman’s correlation coefficient.

Baseline factors associated with albuminuria response were examined using logistic regression. The model included a baseline UACR category (≥30 vs. <30 mg/g), age, sex, baseline eGFR, baseline HbA1c and SGLT2 inhibitor agent, selected a priori as clinically relevant variables available in the dataset; with 60 responders, the number of events per variable was 10. Multicollinearity was assessed using variance inflation factors, calibration with the Hosmer–Lemeshow test and discrimination with the area under the receiver operating characteristic curve (AUC). In the sensitivity analysis, insulin use and hypertension were added. The results are reported as odds ratios (ORs) with 95% CIs. The discriminative ability of baseline UACR alone for albuminuria response was described using the AUC, with a bootstrap 95% CI (2000 resamples); the optimal threshold was identified using the Youden index. A two-sided *p* < 0.05 was considered statistically significant. Given the descriptive aims, no formal sample size calculation or correction for multiple comparisons was performed.

## 3. Results

### 3.1. Baseline Characteristics

The 780 patients had a mean age of 60.3 ± 9.8 years (range 26–89), and 506 (64.9%) were men (Table 1). The mean diabetes duration, baseline HbA1c and baseline eGFR were 12.5 ± 8.0 years (available in 519 patients), 8.6 ± 1.8%, and 92.3 ± 17.8 mL/min/1.73 m^2^, respectively; 39 of 739 patients (5.3%) had a baseline eGFR < 60 mL/min/1.73 m^2^, and CKD was diagnosed in 54 (6.9%). Baseline UACR was available in 327 patients (median 17.0 [8.0–48.0] mg/g), of whom 113 (34.6%) had UACR ≥ 30 mg/g. Hypertension (73.5%) and hyperlipidaemia (77.7%) were common; 80.3% received metformin and 39.5% insulin. Empagliflozin was used in 568 patients (72.8%) and dapagliflozin in 212 (27.2%).

Baseline characteristics were similar between the empagliflozin and dapagliflozin groups for most variables (Table 1). Patients receiving empagliflozin had a lower BMI (32.3 ± 6.4 vs. 33.9 ± 5.8 kg/m^2^, *p* = 0.039); more often, they had a recorded CKD diagnosis (8.3% vs. 3.3%, *p* = 0.023) and less often used metformin (78.5% vs. 85.3%, *p* = 0.042). Baseline HbA1c was numerical, but not significantly higher with empagliflozin (8.7 ± 1.9% vs. 8.5 ± 1.7%, *p* = 0.066).

### 3.2. Changes in Glycaemic, Metabolic and Haematological Parameters

At 12 months, HbA1c decreased by 1.0% (95% CI 0.8–1.1; *n* = 398) and fasting plasma glucose by 37 mg/dL (95% CI 31–44; *n* = 403) (Table 2). Haematocrit increased by 1.3 percentage points (95% CI 1.0–1.7; *n* = 418), uric acid decreased by 0.5 mg/dL (95% CI 0.3–0.7; *n* = 221) and BMI decreased by 1.5 kg/m^2^ (95% CI 0.7–2.2) in the 58 patients with paired values. Triglycerides decreased (mean difference −25 mg/dL; *p* = 0.041), whereas LDL-C changed little (−3.8 mg/dL; paired *t*-test *p* = 0.087; Wilcoxon signed-rank test *p* = 0.034). Serum sodium increased slightly (+0.7 mmol/L) and potassium did not change.

**Figure 2:**
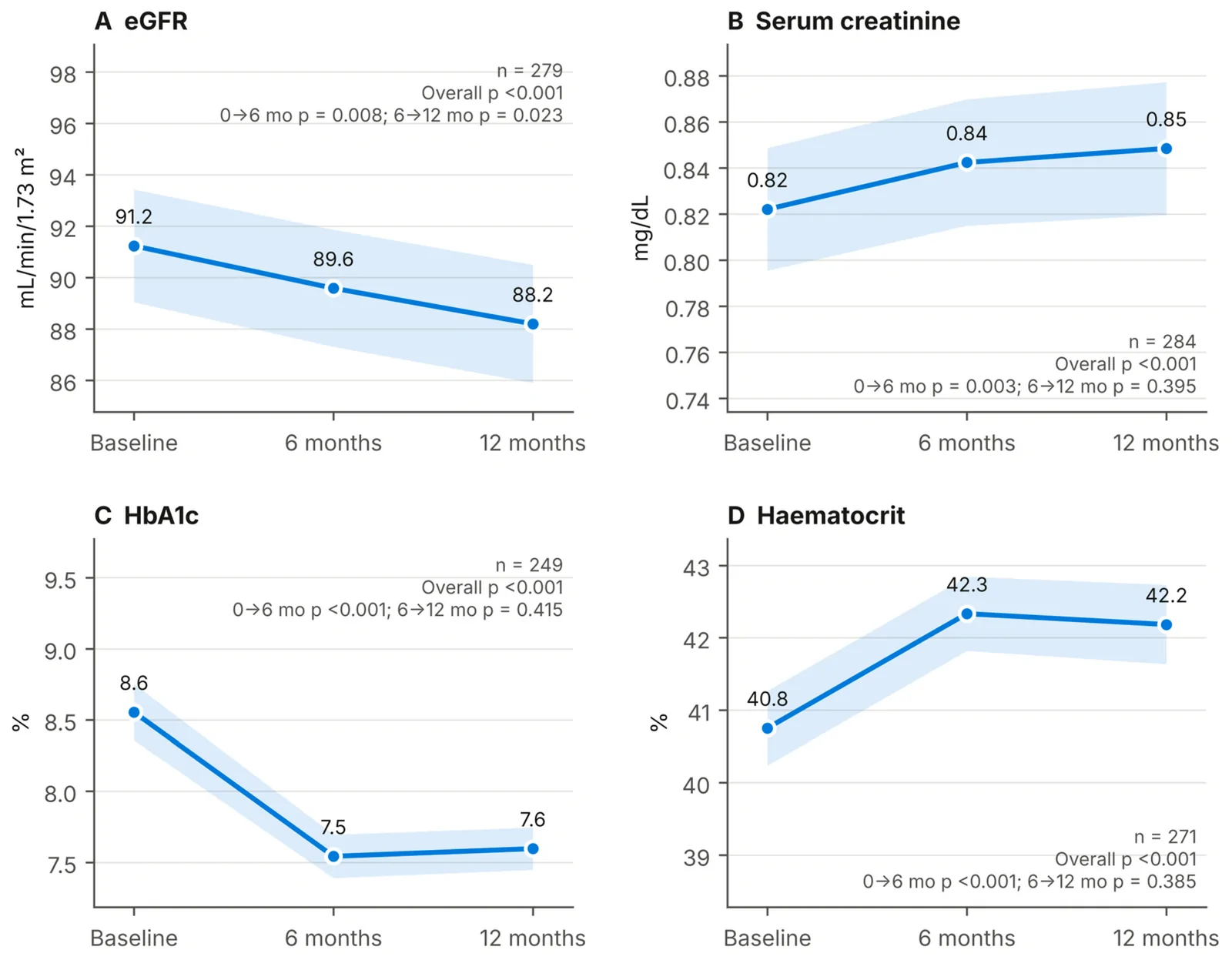
Mean (95% CI) values at baseline, 6 months and 12 months in patients with measurements at all three time points: (**A**) eGFR; (**B**) serum creatinine; (**C**) HbA1c; (**D**) haematocrit. Overall *p* values are derived from the Friedman test; pairwise *p* values are taken from paired *t*-tests. Abbreviations: eGFR, estimated glomerular filtration rate; HbA1c, glycated haemoglobin.

In patients with measurements at all three time points (Figure 2), the HbA1c reduction and the haematocrit increase were largely established by 6 months, with no further significant change between 6 and 12 months (HbA1c *p* = 0.415; haematocrit *p* = 0.385).

### 3.3. Changes in Kidney Function

Mean eGFR decreased by 3.2 mL/min/1.73 m^2^ (95% CI 2.1–4.2; *n* = 426) and serum creatinine increased by 0.03 mg/dL (95% CI 0.02–0.04; *n* = 438) over 12 months (Table 2). Among the 279 patients with eGFR measured at baseline, 6 and 12 months, mean eGFR was 91.2, 89.6 and 88.2 mL/min/1.73 m^2^, respectively (Figure 2A): the mean paired change was −1.7 mL/min/1.73 m^2^ from baseline to 6 months (*p* = 0.008) and −1.4 mL/min/1.73 m^2^ from 6 to 12 months (*p* = 0.023). Serum creatinine increased between baseline and 6 months (*p* = 0.003) and did not change significantly thereafter (*p* = 0.395) (Figure 2B). The decline in eGFR was concentrated in patients with a baseline eGFR ≥90 mL/min/1.73 m^2^ (mean change −4.7 mL/min/1.73 m^2^, *n* = 277); however, it remained essentially unchanged in patients with a baseline eGFR <90 mL/min/1.73 m^2^ (−0.3 mL/min/1.73 m^2^, *n* = 149). A decline in eGFR of ≥30% at 12 months occurred in 13 of 426 patients (3.1%).

### 3.4. Albuminuria

Paired baseline and 12-month UACR values were available in 109 patients (14.0% of the cohort). Compared with the 671 patients without paired UACR data, these patients had similar age, sex distribution, mean baseline eGFR, baseline HbA1c and baseline UACR values (where measured) they were less often treated with empagliflozin (60.6% vs. 74.8%, *p* = 0.003) or pioglitazone (2.8% vs. 14.0%, *p* < 0.001) and more likely to receive insulin (54.2% vs. 37.1%, *p* = 0.001) or a GLP-1 receptor agonist (8.3% vs. 3.3%, *p* = 0.028). A greater number of patients had hyperlipidaemia (86.2% vs. 76.3%, *p* = 0.029) or a baseline eGFR <60 mL/min/1.73 m^2^ (10.1% vs. 4.4%, *p* = 0.028).

**Figure 3:**
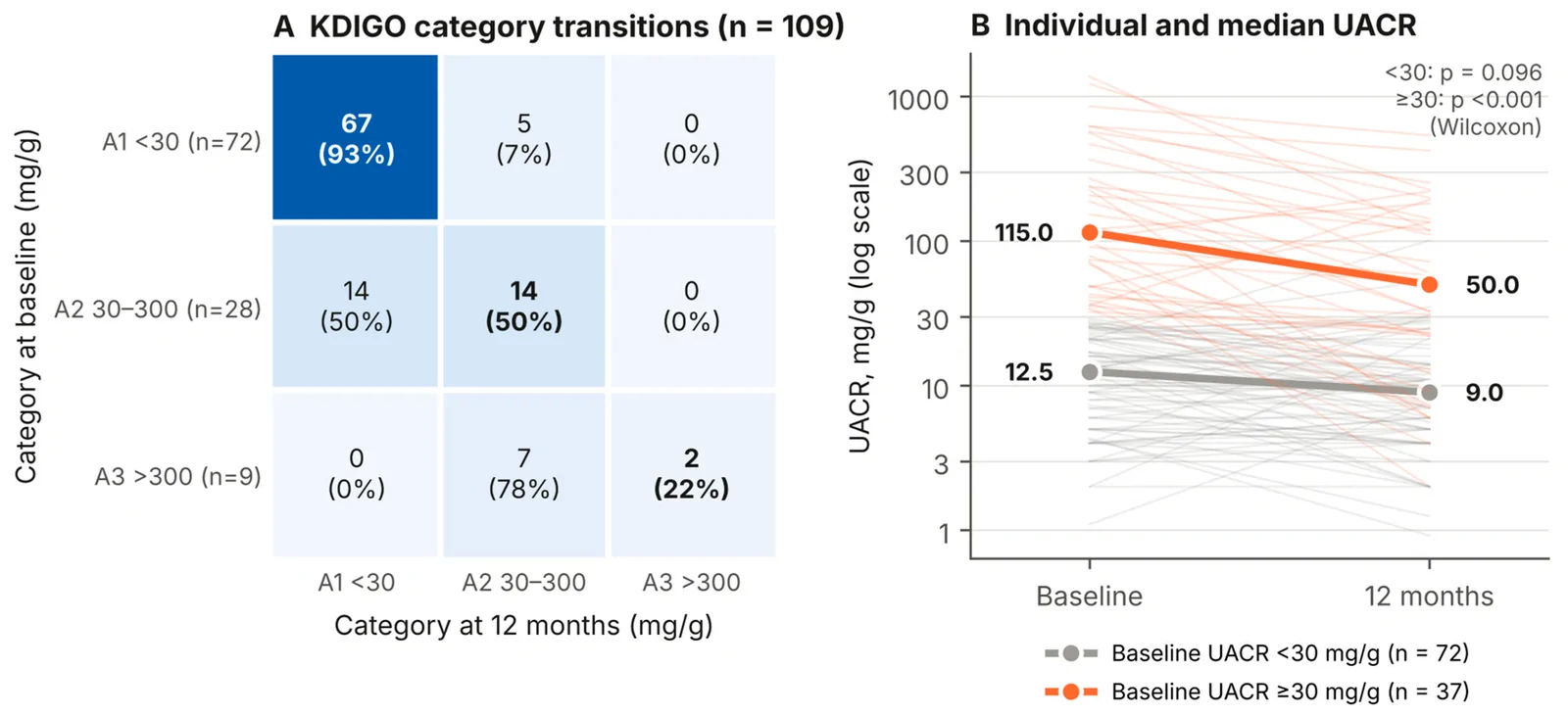
Albuminuria in patients with paired baseline and 12-month UACRs (*n* = 109). (**A**) Transitions between KDIGO albuminuria categories: cells show the number of patients (row percentage); the diagonal indicates no change in category. (**B**) Individual (thin lines) and median (thick lines) UACRs by baseline category; *p* values are derived from the Wilcoxon signed-rank test. Abbreviations: KDIGO, Kidney Disease: Improving Global Outcomes; UACR, urinary albumin-to-creatinine ratio.

In these 109 patients, median UACR decreased from 20.9 [9.0–49.0] mg/g at baseline to 11.0 [6.0–30.0] mg/g at 12 months (*p* < 0.001, Wilcoxon signed-rank test; Table 3). The geometric mean ratio corresponded to a 38.8% reduction (95% CI 25.0–50.1%), which was larger in the 37 patients with baseline UACR ≥30 mg/g (median 115.0 [49.0–270.0] to 50.0 [22.0–135.6] mg/g; median relative change −66.7%; *p* < 0.001) than in the 72 patients with baseline UACR <30 mg/g (12.5 [5.0–20.6] to 9.0 [5.0–14.0] mg/g; median relative change −19.4%; *p* = 0.096) (Figure 3B). Of the 28 patients with A2 albuminuria at baseline, 14 (50.0%) were in category A1 at 12 months; of the 9 patients with A3 albuminuria, 7 (77.8%) were in category A2. Conversely, 5 of the 72 patients (6.9%) with A1 albuminuria at baseline were in category A2 at 12 months (Figure 3A). In the 51 patients with UACR at all three time points, the median UACR was 25.9, 14.2 and 13.8 mg/g at baseline, 6 and 12 months, respectively (Friedman *p* < 0.001).

**Figure 4:**
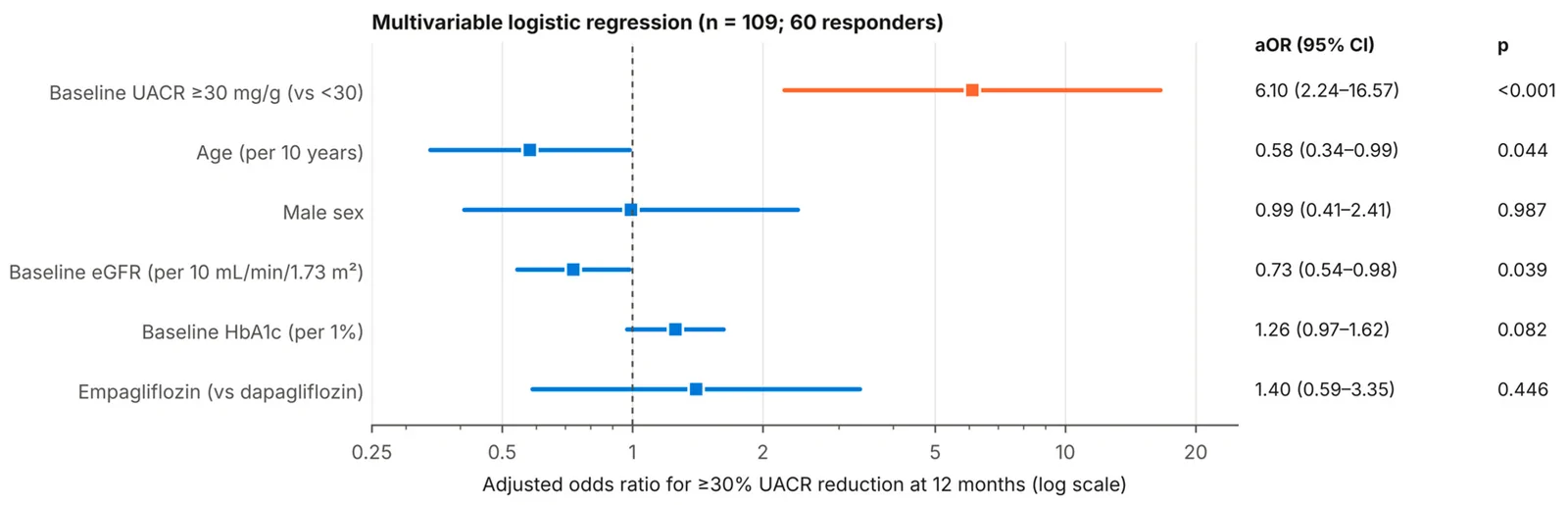
Baseline factors associated with albuminuria response (≥30% reduction in UACR at 12 months) in multivariable logistic regression (*n* = 109; 60 responders). Squares indicate adjusted odds ratios and lines show 95% confidence intervals. Abbreviations: aOR, adjusted odds ratio; CI, confidence interval; eGFR, estimated glomerular filtration rate; UACR, urinary albumin-to-creatinine ratio.

Albuminuria response (≥30% UACR reduction) occurred in 60 of 109 patients (55.0%), including 30 of 37 (81.1%) with a baseline UACR ≥ 30 mg/g and 30 of 72 (41.7%) with a baseline UACR < 30 mg/g (unadjusted OR 6.00, 95% CI 2.33–15.46). In multivariable analysis, a baseline UACR ≥ 30 mg/g was associated with albuminuria response (adjusted OR 6.10, 95% CI 2.24–16.57; *p* < 0.001), alongside younger age (OR 0.58 per 10 years, 95% CI 0.34–0.99) and lower baseline eGFR (OR 0.73 per 10 mL/min/1.73 m^2^, 95% CI 0.54–0.98) (Figure 4). The model showed no relevant multicollinearity (all variance inflation factors were ≤1.34), acceptable calibration (Hosmer–Lemeshow *p* = 0.24) and an AUC of 0.78. The association was similar after additional adjustment for insulin use and hypertension (adjusted OR 7.13, 95% CI 2.48–20.46; *n* = 107). Baseline UACR discriminated responders from non-responders with an AUC of 0.80 (95% CI 0.71–0.87); the Youden-optimal threshold was 14 mg/g (sensitivity 83.3%, specificity 61.2%). Changes in the UACR and eGFR were not correlated (Spearman’s ρ = 0.04, *p* = 0.71; *n* = 108).

### 3.5. Empagliflozin Versus Dapagliflozin

Twelve-month changes in HbA1c, eGFR, serum creatinine, haematocrit, uric acid and UACR did not differ significantly between patients treated with empagliflozin and dapagliflozin (all *p* > 0.05). The study was not designed or powered to compare the two agents, and the confidence intervals were wide.

### 3.6. Safety and Treatment Discontinuation

Urinary tract and genital mycotic infections were recorded in 106 (13.6%) and 46 (5.9%) patients, respectively (Table 4), and were both more frequent in women than in men (urinary tract infection 29.3% vs. 5.1%; genital infection 15.4% vs. 0.8%; both *p* < 0.001). SGLT2 inhibitor treatment was discontinued in 116 patients (14.9%), with women ceasing treatment in more cases than men (23.4% vs. 10.3%, *p* < 0.001). Among the 103 patients with a documented reason, discontinuation was caused by a urinary tract infection in 51 (49.5%), genital infection in 6 (5.8%), gastrointestinal complaints in 8 (7.8%), allergy in 2 (1.9%) and other reasons in 36 (35.0%). Diabetic ketoacidosis, volume depletion, acute kidney injury and hypoglycaemia were not systematically documented and could not be assessed.

## 4. Discussion

This retrospective cohort comprised 780 adults with T2DM who started treatment with empagliflozin or dapagliflozin in routine care at a Turkish university hospital. The first year of treatment was accompanied by an approximately 1% reduction in HbA1c, a 1.3 percentage point increase in haematocrit, a 0.5 mg/dL reduction in uric acid, and a modest eGFR decline of about 3 mL/min/1.73 m^2^. In the subgroup with paired UACR measurements, the median UACR fell by almost half, with larger reductions in patients with baseline albuminuria. Because this study lacked a comparator group, these findings describe changes observed after treatment initiation and cannot be attributed to SGLT2 inhibition alone.

The direction of these changes is consistent with randomised trials, although their magnitude should be compared with caution. The geometric mean UACR reduction of 39% in our subgroup and the decrease in the median UACR of approximately 47% (from 20.9 to 11.0 mg/g) are at or above the upper end of the placebo-adjusted reductions reported in trials, such as 25% and 32% in patients with micro- and macroalbuminuria in EMPA-REG OUTCOME [14] and 35% in patients with T2DM in DAPA-CKD [17], and above the pooled estimate of a meta-analysis of randomised trials [18]. These estimates are not directly comparable, and several factors may have inflated our within-patient estimate. First, our estimate is based on a within-patient change without a placebo arm; it therefore includes changes that would have occurred without SGLT2 inhibition, including the effects of concomitant therapy and improved glycaemic control. Second, UACR has substantial within-person variability [29]; patients may have been selected for testing due to a previously elevated value, which leads to a regression to the mean [30]. Third, only 109 of 780 patients (14.0%) had paired UACR values, and these patients differed from the rest of the cohort in some treatment characteristics. The UACR estimates should therefore be regarded as descriptive and hypothesis-generating.

The strong association between baseline albuminuria ≥ 30 mg/g and a ≥30% UACR reduction (adjusted OR 6.10) must be interpreted cautiously. Patients with normoalbuminuria have less room for a clinically meaningful reduction (a floor effect), and patients with high initial values are most susceptible to a regression in the mean; both mechanisms favour greater apparent responses in those with higher baseline values independently of any drug effect. Nevertheless, this pattern is consistent with trial analyses showing larger absolute UACR reductions with SGLT2 inhibitors at higher baseline albuminuria [14,15,16,17]. Our data do not imply that patients with UACR < 30 mg/g do not receive any benefits from such treatment: cardiovascular and kidney risk rises continuously from UACR levels well below 30 mg/g [3], and the benefits of SGLT2 inhibitors related to this in trials were not confined to patients with albuminuria [7,9,13]. Associations with younger age and lower baseline eGFR were modest, though they were not pre-specified hypotheses and require confirmation.

The mean eGFR decline of 3.2 mL/min/1.73 m^2^ over 12 months should not be interpreted as evidence of the classic initial haemodynamic “dip”, which occurs within the first 2–4 weeks of treatment during trials [20]. By contrast, the earliest follow-up measurement was at 6 months. In a recent single-centre study from Türkiye in which eGFR was measured 14–90 days after SGLT2 inhibitor initiation, an early eGFR decline was observed in 76% of patients, alongside reductions in albuminuria [31]. About half of the 12-month decline had already occurred at 6 months, with a further smaller decline between 6 and 12 months; serum creatinine rose between baseline and 6 months and then remained stable. This pattern is compatible with an early haemodynamic effect; however, without early measurements or a control group, it cannot be distinguished from the decline expected with ageing and diabetes. The age-related factor of the CKD-EPI equation alone accounts for a decrease of approximately 0.6–0.7 mL/min/1.73 m^2^ per year at this eGFR level, even when serum creatinine is constant [27]. The decline was concentrated in patients with a baseline eGFR ≥ 90 mL/min/1.73 m^2^, which may indicate attenuation of glomerular hyperfiltration but may also reflect a regression in the mean for patients with high initial values. The small increase in serum creatinine was concordant with the decrease in eGFR. The absence of a correlation between the changes in eGFR and UACR suggests that variations in albuminuria in this cohort were not simply a consequence of differences in filtration.

The rise in haematocrit, evident at 6 months and stable thereafter, is consistent with trial observations [21]. Two mechanisms have been proposed to explain this: haemoconcentration due to osmotic diuresis and natriuresis, and increased erythropoietin production, possibly related to reductions in tubular oxygen demand and altered nutrient-deprivation and hypoxia signalling [22,32]. Haemoglobin, erythropoietin, reticulocytes, iron indices and plasma volume were not measured in our study; therefore, we cannot distinguish between these mechanisms. Similarly, the reduction in uric acid agrees with the urate-lowering effect of SGLT2 inhibitors reported in a meta-analysis of randomised trials [23], and the reduction in triglycerides and the minimal change in LDL-C are compatible with pooled trial data [33]. However, improved glycaemic control and changes in concomitant therapy may also have contributed to these differences.

Genitourinary infections were the main recorded adverse events and the most common reason for discontinuation. The rates in women (29.3% for urinary tract infection and 15.4% for genital infection) are higher than those reported in randomised trials [34]. Our definition relied on documented clinical diagnoses and prescriptions over a median treatment period longer than 1 year, without culture confirmation; therefore, our data could include episodes unrelated to treatment and recurrent infections in patients with a prior history. As such, these rates are not directly comparable with trial-adjudicated events. Diabetic ketoacidosis, volume depletion, acute kidney injury and hypoglycaemia were not systematically documented and could not be evaluated.

Large real-world studies have associated SGLT2 inhibitor use with lower risks of heart failure hospitalisation and death compared with other glucose-lowering drugs [24,25]. Unlike studies using comparator groups and clinical endpoints, our research describes laboratory changes in a single centre. Its contribution lies in documenting the characteristics and 1-year laboratory changes in patients starting treatment with SGLT2 inhibitors in routine Turkish practice, including the frequency of UACR testing and genitourinary infections leading to discontinuation.

### Limitations

This study has important limitations. First, it was a single-centre retrospective study without a control group of patients not receiving SGLT2 inhibitors. As such, treatment was not randomly allocated; the observed changes are associations and cannot be entirely attributed to SGLT2 inhibition. Second, 979 of 1759 screened patients were excluded because their records were not retrievable; as such, their characteristics could not be compared to the included patients, making selection bias possible. Third, laboratory data were incomplete: each analysis used a different subset of patients, and paired UACR values were available in only 14.0% of the cohort. Patients with UACR data differed from the rest of the cohort in some treatment characteristics, and we cannot determine why the UACR was measured; reduced outpatient attendance during the COVID-19 pandemic and incomplete UACR screening in routine practice, as reported elsewhere [5], may have contributed to this. Fourth, the analysis was restricted to patients who continued treatment for at least 12 months; patients who discontinued earlier, with greater susceptibility to adverse events or different laboratory trajectories, were not represented, and an intention-to-treat analysis was not possible. Fifth, data on blood pressure, renin–angiotensin system inhibitors, diuretics, mineralocorticoid receptor antagonists, SGLT2 inhibitor dose, changes in concomitant medication during follow-up, adherence and haemoglobin were not available; residual confounding by these and other unmeasured factors cannot be excluded. Sixth, measurements were available only at baseline, 6 and 12 months; early haemodynamic eGFR response could not be assessed, and single UACR values per time point are subject to considerable within-person variability. Seventh, eGFR was estimated with the 2009 CKD-EPI equation used by the laboratory during the study period; using the 2021 race-free equation would yield slightly different values. Eighth, the comparison of empagliflozin and dapagliflozin was underpowered and reflects local prescribing patterns. Finally, follow-up was limited to a period of 1 year, which is sufficient to capture laboratory responses to SGLT2 inhibition that develop within weeks to months of treatment initiation [14,20,21]. Effects were largely established by 6 months in our cohort. For instance, short-term reductions in UACR have been associated with a lower long-term risk of cardiovascular and kidney outcomes [28,35]. However, no clinical kidney or cardiovascular endpoints were assessed, and the observed changes in surrogate markers do not establish long-term kidney protection.

## 5. Conclusions

In adults with T2DM who started treatment with empagliflozin or dapagliflozin in routine care, the first year of treatment was accompanied by reductions in HbA1c, uric acid and albuminuria, an increase in haematocrit and a modest decline in eGFR, changes that are similar in direction to those reported in randomised trials. Larger albuminuria reductions in patients with baseline UACR ≥30 mg/g are likely to reflect, at least in part, floor effects and a regression to the mean. Given the uncontrolled design and incomplete laboratory data, these findings are descriptive; prospective studies with comparator groups and systematic UACR measurements are needed to evaluate the effects of SGLT2 inhibitors in this population.

## Figures and Tables

**Table 1:** Baseline characteristics of the study cohort: overall and by SGLT2 inhibitor agent.

Characteristic	*n* with Data	Overall (*n* = 780)	Empagliflozin (*n* = 568)	Dapagliflozin (*n* = 212)	*p*
Age, years	780	60.3 ± 9.8	60.3 ± 9.8	60.5 ± 9.8	0.807
Diabetes duration, years	519	12.5 ± 8.0	12.4 ± 8.3	12.7 ± 7.1	0.602
BMI, kg/m^2^	268	32.8 ± 6.2	32.3 ± 6.4	33.9 ± 5.8	0.039
HbA1c, %	746	8.6 ± 1.8	8.7 ± 1.9	8.5 ± 1.7	0.066
Fasting plasma glucose, mg/dL	722	178 ± 65	180 ± 67	173 ± 58	0.197
Serum creatinine, mg/dL ^a^	753	0.79 [0.68–0.93]	0.80 [0.69–0.94]	0.78 [0.67–0.90]	0.141
eGFR, mL/min/1.73 m^2^	739	92.3 ± 17.8	91.5 ± 17.8	94.4 ± 17.8	0.051
UACR, mg/g ^a^	327	17.0 [8.0–48.0]	16.9 [6.7–49.0]	17.2 [9.0–43.1]	0.563
Haematocrit, %	730	41.4 ± 4.3	41.4 ± 4.3	41.5 ± 4.1	0.673
LDL-C, mg/dL	674	122.2 ± 41.3	122.5 ± 40.9	121.3 ± 42.4	0.732
Triglycerides, mg/dL ^a^	673	176 [123.0–258.0]	175 [123.0–248.0]	182 [122.0–264.0]	0.568
Uric acid, mg/dL	550	5.6 ± 1.5	5.6 ± 1.4	5.7 ± 1.5	0.408
Male sex, *n* (%)	780	506 (64.9)	367 (64.6)	139 (65.6)	0.870
Hypertension, *n* (%)	780	573 (73.5)	420 (73.9)	153 (72.2)	0.683
Hyperlipidaemia, *n* (%)	780	606 (77.7)	442 (77.8)	164 (77.4)	0.968
Coronary artery disease, *n* (%)	780	295 (37.8)	227 (40.0)	68 (32.1)	0.053
Heart failure, *n* (%)	780	69 (8.8)	55 (9.7)	14 (6.6)	0.228
Cerebrovascular disease, *n* (%)	780	22 (2.8)	19 (3.3)	3 (1.4)	0.222
Peripheral artery disease, *n* (%)	780	23 (2.9)	18 (3.2)	5 (2.4)	0.721
Chronic kidney disease (recorded diagnosis), *n* (%)	780	54 (6.9)	47 (8.3)	7 (3.3)	0.023
Malignancy (history), *n* (%)	780	60 (7.7)	45 (7.9)	15 (7.1)	0.807
Diabetic foot, *n* (%)	780	17 (2.2)	16 (2.8)	1 (0.5)	0.052
Metformin, *n* (%)	773	621 (80.3)	441 (78.5)	180 (85.3)	0.042
Sulfonylurea, *n* (%)	773	129 (16.7)	92 (16.4)	37 (17.5)	0.780
Pioglitazone, *n* (%)	773	96 (12.4)	65 (11.6)	31 (14.7)	0.293
DPP-4 inhibitor, *n* (%)	773	303 (39.2)	212 (37.7)	91 (43.1)	0.197
GLP-1 receptor agonist, *n* (%)	772	31 (4.0)	23 (4.1)	8 (3.8)	1.000
Insulin (any), *n* (%)	772	305 (39.5)	217 (38.6)	88 (41.9)	0.453
Baseline eGFR <60 mL/min/1.73 m^2^, *n* (%)	739	39 (5.3)	28 (5.2)	11 (5.6)	0.954
Baseline UACR ≥30 mg/g, *n* (%)	327	113 (34.6)	79 (34.3)	34 (35.1)	1.000

Values are the mean ± SD, median [IQR] (^a^) or *n* (%); percentages are calculated among patients with available data. *p* values compare empagliflozin with dapagliflozin (Welch’s *t*-test, Mann–Whitney U test, chi-squared or Fisher’s exact test). Blood pressure, renin–angiotensin system inhibitor and diuretic use were not available in the dataset. Abbreviations: BMI, body mass index; CKD, chronic kidney disease; DPP-4, dipeptidyl peptidase-4; eGFR, estimated glomerular filtration rate; GLP-1, glucagon-like peptide-1; HbA1c, glycated haemoglobin; LDL-C, low-density lipoprotein cholesterol; UACR, urinary albumin-to-creatinine ratio.

**Table 2:** Changes in laboratory parameters from baseline to 12 months in patients with paired measurements.

Parameter	*n*	Baseline	12 Months	Mean Change (95% CI)	*p*, *t*-Test	*p*, Wilcoxon
HbA1c, %	398	8.7 ± 1.7	7.7 ± 1.4	−1.0 (−1.1 to −0.8)	<0.001	<0.001
Fasting plasma glucose, mg/dL	403	179 ± 65	141 ± 42	−37 (−44 to −31)	<0.001	<0.001
BMI, kg/m^2^	58	33.0 ± 7.0	31.5 ± 6.6	−1.5 (−2.2 to −0.7)	<0.001	<0.001
Serum creatinine, mg/dL	438	0.83 ± 0.23	0.85 ± 0.25	+0.03 (0.02 to 0.04)	<0.001	<0.001
eGFR, mL/min/1.73 m^2^	426	91.9 ± 18.0	88.7 ± 19.0	−3.2 (−4.2 to −2.1)	<0.001	<0.001
Haematocrit, %	418	41.0 ± 4.3	42.3 ± 4.6	+1.3 (1.0 to 1.7)	<0.001	<0.001
Uric acid, mg/dL	221	5.6 ± 1.5	5.1 ± 1.4	−0.5 (−0.7 to −0.3)	<0.001	<0.001
LDL-C, mg/dL	339	119.0 ± 39.5	115.2 ± 42.1	−3.8 (−8.1 to 0.6)	0.087	0.034
Triglycerides, mg/dL	341	229 ± 224	203 ± 192	−25 (−49 to −1)	0.041	<0.001
Sodium, mmol/L	353	138.9 ± 2.7	139.6 ± 2.4	+0.7 (0.4 to 1.0)	<0.001	<0.001
Potassium, mmol/L	359	4.50 ± 0.42	4.48 ± 0.41	−0.02 (−0.07 to 0.03)	0.402	0.625
UACR, mg/g ^a^	109	20.9 [9.0–49.0]	11.0 [6.0–30.0]	GMR −38.8% (−50.1 to −25.0)	<0.001	<0.001

Values are mean ± SD unless otherwise stated. Each row includes all patients with both baseline and 12-month values for that parameter. ^a^ UACR is presented as the median [IQR]; the change is the geometric mean ratio (GMR) expressed as percentage change (95% CI), and the *t*-test *p* value is from the paired *t*-test on log-transformed values. Abbreviations are the same as Table 1.

**Table 3:** Urinary albumin-to-creatinine ratio at baseline and 12 months alongside albuminuria outcomes (*n* = 109).

	Baseline	12 Months		*p*
UACR, all patients				
*n*	109			
Median [IQR], mg/g	20.9 [9.0–49.0]	11.0 [6.0–30.0]		<0.001
UACR <30 mg/g at baseline (A1)				
*n*	72			
Median [IQR], mg/g	12.5 [5.0–20.6]	9.0 [5.0–14.0]		0.096
UACR ≥30 mg/g at baseline (A2–A3)				
*n*	37			
Median [IQR], mg/g	115.0 [49.0–270.0]	50.0 [22.0–135.6]		<0.001
Outcome at 12 months			*n* (%)	
≥30% UACR reduction, all patients			60 (55.0)	
≥30% UACR reduction, baseline UACR <30 mg/g (*n* = 72)			30 (41.7)	
≥30% UACR reduction, baseline UACR ≥30 mg/g (*n* = 37)			30 (81.1)	
Regression from A2 to A1 (*n* = 28)			14 (50.0)	
Regression from A3 to A2 (*n* = 9)			7 (77.8)	
Progression from A1 to A2 (*n* = 72)			5 (6.9)	

*p* values are derived from the Wilcoxon signed-rank test. KDIGO albuminuria categories: A1 < 30 mg/g; A2 30–300 mg/g; A3 > 300 mg/g. Abbreviations: IQR; interquartile range; UACR; urinary albumin-to-creatinine ratio.

**Table 4:** Genitourinary infections and treatment discontinuation by sex.

Event	All (*n* = 780)	Men (*n* = 506)	Women (*n* = 274)		*p*
Urinary tract infection	106 (13.6)	26 (5.1)	80 (29.3)		<0.001
Genital mycotic infection	46 (5.9)	4 (0.8)	42 (15.4)		<0.001
Treatment discontinuation	116 (14.9)	52 (10.3)	64 (23.4)		<0.001
Reason for discontinuation (*n* = 103 with documented reason)				***n* (%)**	
Urinary tract infection				51 (49.5)	
Genital infection				6 (5.8)	
Gastrointestinal complaints				8 (7.8)	
Allergy				2 (1.9)	
Other				36 (35.0)	

Values are given as *n* (%). Infection status was missing for 1 patient. Infections were defined as clinically diagnosed and treated episodes documented in outpatient notes and prescription records. Diabetic ketoacidosis, volume depletion, acute kidney injury and hypoglycaemia were not systematically recorded.

## Data Availability

The data presented in this study are available from the corresponding author upon reasonable request. The data are not publicly available because of patient privacy and ethical restrictions.

## Abbreviations

The following abbreviations are used in this manuscript: aOR, adjusted odds ratio; AUC, area under the curve; BMI, body mass index; CI, confidence interval; CKD, chronic kidney disease; CKD-EPI, Chronic Kidney Disease Epidemiology Collaboration; DKD, diabetic kidney disease; DPP-4, dipeptidyl peptidase-4; eGFR, estimated glomerular filtration rate; GLP-1, glucagon-like peptide-1; GMR, geometric mean ratio; HbA1c, glycated haemoglobin; IQR, interquartile range; KDIGO, Kidney Disease: Improving Global Outcomes; LDL-C, low-density lipoprotein cholesterol; OR, odds ratio; SD, standard deviation; SGLT2, sodium–glucose co-transporter 2; T2DM, type 2 diabetes mellitus; UACR, urinary albumin-to-creatinine ratio.
